# Emergent Intraverbal and Reverse Intraverbal Behavior Following Listener Training in Children with Autism Spectrum Disorder

**DOI:** 10.1007/s40616-021-00164-3

**Published:** 2022-01-11

**Authors:** Inger Karin Almås, Dean P. Smith, Sigmund Eldevik, Svein Eikeseth

**Affiliations:** 1 Department of Behavioral Science, Oslo Metropolitan University, P. O. Box 4, St. Olavs Plass, N-0130 Oslo, Norway; 2 UK Young Autism Project, London, UK

**Keywords:** intraverbal behavior, emergent intraverbal behavior, reverse intraverbal behavior, listener training, autism

## Abstract

We evaluated whether intraverbal and reverse intraverbal behavior emerged following listener training in children with autism spectrum disorder (ASD). Six participants were each taught three sets of three “when?” questions in listener training. A multiple baseline design across behaviors (stimulus sets) was used to assess the effects of listener training. Results showed that intraverbal behavior emerged following listener training for five out of six participants. One participant received additional listener training and intraverbal training before intraverbal behavior emerged. Furthermore, reverse intraverbal responding occurred across all three sets of questions for three of the six participants. Establishing listener behavior may be a pathway for emergent intraverbal and reverse intraverbal responding in children with ASD. Future research could examine what skill repertoire may facilitate such transfer.

Children with autism spectrum disorder (ASD) often show deficits in acquiring intraverbal behavior (Sundberg & Sundberg, 2011), defined as verbal responses that do not share point-to-point correspondence with their verbal antecedents (Skinner, 1957; Sundberg, 2016). Whilst typically developing children learn intraverbal forms in their preschool years (e.g., answering simple social questions at 2 years of age, Sundberg, 2008), children with ASD may develop no intraverbal repertoire or only limited intraverbal behavior. These difficulties may arise because some children with ASD do not learn simple, conditional, and/or compound discriminations, especially when the antecedent stimuli are verbal (Axe, 2008; Eikeseth, & Smith, 2013; Sundberg, 2016; Sundberg & Sundberg, 2011). The focus of some research has been to directly teach the intraverbal response using a vocal (echoic) prompt (e.g., Xu et al., 2020; Ingvarsson & Hollobaugh, 2010), a picture (tact) prompt (e.g., Devine et al., 2016; Miguel et al., 2005), a written (textual) prompt (e.g., Vedora et al., 2009) or a speech-generating device (Carnett et al., 2017). The prompt is then faded to transfer stimulus control from the prompt to the verbal antecedent stimulus (i.e., to the question).

Intraverbal behavior can also be established without direct training, such as when emerging following tact training, listener training, or after learning other intraverbal responses (DeSouza et al., 2017). In fact, the existence of an advanced tact and listener repertoire is, according to Sundberg and Sundberg (2011), the foundation for an advanced intraverbal repertoire. Smith et al. (2016) examined the effectiveness of listener training on emergent intraverbal behavior in five participants with ASD, who already were able to tact the stimuli involved in the listener training. Intraverbal behavior took the form of answers to questions about function. Each target question was taught first as a listener response to which the participant was required to touch the correct picture (i.e., during listener training the participant was asked, “what do you eat that’s yellow?” and the participant touched the picture of the banana). Following listener training, probe sessions were conducted in which participants were asked the same question but in the absence of the picture to assess emergence of the intraverbal relation. Results showed the emergence of intraverbal behavior for four of the five participants. One participant was taught to tact the picture as part of the listener response before emergence of intraverbal behavior occurred. The participants in this study were able to tact all pictures used during listener training before participation in the study.

Kodak and Paden (2015) examined whether listener training facilitated the acquisition of intraverbal behavior in two children with ASD. During listener training, the experimenter placed visual stimuli in front of the participant and presented an antecedent verbal stimulus (e.g., /You wash with ___/), and the participants were trained to touch the correct visual stimuli. During probes for emergent intraverbal behavior, the same verbal antecedent stimuli were presented without the visual stimuli. The results showed that listener training led to the emergence of intraverbal behavior for only one of the two participants. Hence, even though listener training can lead to emergent intraverbal behavior for some participants with ASD, it is not always effective. Similar findings have been reported in studies with typically developing children (Cortez et al., 2020; Lechago et al., 2015; Petursdottir & Haflidadottir, 2009; Petursdottir et al., 2008b; Petursdottir et al., 2014).

Given the conflicting results and the fact that there are only two previous studies reporting on emergent intraverbal responding following listener training in individuals with ASD, the current study was designed as a systematic replication of Smith et al. (2016). We extended Smith et al. by examining whether listener training could result in a second type of emergent intraverbal responding, namely emergence of reverse intraverbal responding. In reverse intraverbal responding, the B-A relation emerges following training of an A-B relation (Pérez-González et al., 2007; Pérez-González et al., 2018). For example, the instruction, “name the opposite of thick” (answer, “thin”) can be reversed to, “name the opposite of thin” (answer, “thick”). There is seemingly no difference between which is taught, and which emerges, and the elements “thick” and “thin” are present in both either as an antecedent or as a response (Pérez-González et al., 2018). Bidirectional or reverse relations are present beyond intraverbal behavior and throughout other verbal behavior, such as naming (Horne & Lowe, 1996) and categorization (Pérez-González et al., 2018), as well as being a property of stimulus equivalence and relational frames (Hayes et al., 2001; Miguel, 2016; Jennings & Miguel, 2017; Sidman & Tailby, 1982).

Although, previous studies have implemented listener training and probed for the emergence of reverse intraverbals in typically developing children (e.g., Cortez et al., 2020; Petursdottir et al., 2008b), only three studies have examined the emergence of reverse intraverbal responding in children with ASD (Allan et al., 2015; Dickes & Kodak, 2015; Thakore & Petursdottir, 2021). In Allan et al. (2015), intraverbal training included multiple exemplars taught concurrently, constant prompt-delay procedures, programmed reinforcement, and bidirectional stimulus-response teaching formats. Three of four participants showed emergence of reverse intraverbals following intraverbal training. Thakore and Petursdottir (2021) examined the effects of intraverbal instruction using fluency training on the acquisition of divergent intraverbal responding in two children with ASD. Teaching with tact prompts produced only small increases in intraverbal responding, whereas the addition of fluency training quickly produced criterion-level performance. Both participants demonstrated generalization to untrained questions including some reverse intraverbals.

To date, no studies have examined the extent to which reverse intraverbal responding emerges from listener training in children with ASD. Possibly, bidirectional intraverbal relations may occur as a result of listener training. For example, a child may learn to answer the question, /When do you use toothpaste?/ with the vocal response, “When brushing teeth” after listener training in which the child receives reinforcers for touching the picture of brushing teeth when hearing the question, /When do you use toothpaste?/. Emitting the reverse intraverbal response in this case would require saying, “Toothpaste” when asked, /What do you put on the toothbrush when you brush your teeth?/

If listener training can result in the emergence of intraverbals in both directions, then the instruction is not only effective but also most efficient. Also, this could provide a demonstration of how language, in this case intraverbals, occur in the absence of direct training. Indeed, research examining the emergence of relations that are not directly taught is important for at least two reasons; firstly, it provides a procedure for an efficient teaching strategy, and secondly, it shows how behavior analysts may account for verbal behavior emerging without a direct reinforcement history. Because children seem to acquire much language without direct teaching, behavior analysts need to account for this, both empirically and conceptually.

## Method

### Participants, Setting and Materials

Six boys diagnosed with ASD participated in this study. Participant’s age ranged from 4 to 11 years. All participants attended mainstream school and were receiving intensive behavioral intervention programs at school. Table 1 shows the age and percent correct performance on subsections of the Assessment of Basic Language and Learning Skills – Revised (ABLLS-R; Partington, 2010) prior to the study. Table 2 shows participants' prerequisite skills for learning intraverbals as scored on the ABLLS-R.

**Table 1:** Age in Years and Percent Correct Performance on Subsections of the Assessment of Basic Language and Learning Skills (ABLLS-R; Partington, 2010) for each Participant Prior to the Study

	P 1 _____	P 2 _____	P 3 _____	P 4 _____	P 5 _____	P 6 _____
Age (years)	9	10	11	11	4	5
Visual performance	90%	75%	90%	-	-	-
Receptive language	81%	85%	76%	-	85%	96%
Vocal imitation	95%	90%	75%	-	25%	100%
Requesting	6%	46%	29%	-	76%	55%
Labeling	22%	51%	38%	-	76%	61%
Intraverbals	26%	21%	17%	-	22%	45%
Spontaneous Vocalizations	93%	72%	82%	-	100%	100%

Data for Participant 4 and Visual Performance Data for Participants 5 and 6 were not Available

*Note: P = Participant.*

**Table 2:** Participants' Prerequisite Skills for Learning Intraverbals as Scored on The Assessment of Basic Language and Learning Skills – Revised (ABLLS-R; Partington, 2010)

Task and Task Name	P 1	P 2	P 3	P 5	P6
H1: Fill in Words from Songs	3 Words/3 Songs	0 words/3 songs	≥3 phrases/6 songs	≥3 phrases/6 songs	≥3 phrases/≥3 phrases/6 songs
	3/4	0/4	4/4	4/4	4/4
H4: Saying Animal Name when Hearing AnimalSound	≥8	≥8	≥8	≥8	≥8
	4/4	4/4	4/4	4/4	4/4
H11: Answering What Questions about Functions of Objects	10	25	10	5	10
	2/4	3/4	2/4	1/4	2/4
H24: Answering What Questions about Activities Community	5	5	0	10	2
	2/4	2/4	0/4	2/4	1/4
H27: Fill in Class when Told two or More Items from the Class	5	0	0	10	1
	2/4	0/4	0/4	3/4	1/4
H28: Answering Who/Whose Questions	1/4	1/4	0/4	1/4	1/4
H29: Answering When Questions	0	0	0	0	5
		0/4	0/4	0/4	0/4
H33; Answering Why Questions	0	0	0	10	5
	0/4	0/4	0/4	2/4	1/4

*Note: P = Participant. 0/4 = a raw score of 0 out of 4; 1/4 = a raw score of 1 out of 4; 2/4 = a raw score of 2 out of 4; 3/4 = a raw score of 3 out of 4; 4/4 = a raw score of 4 out of 4. Data for Participant 4 were not Available.*

The participants were able to tact all picture stimuli used during listener training. All picture stimuli were taken from online sources such as Google Images^TM^ and were included because they were deemed to be clear depictions of the intraverbal response. Pictures were in color and presented as individual cards (size ranged from 5 cm x 4 cm to 10 cm x 7 cm). Table 3 shows an example of sample and comparison stimuli used during listener training, and the verbal antecedent and response used during intraverbal and reverse intraverbal probes. All sessions were conducted at the participants’ school or kindergarten in a separate room containing a table and chairs and teaching materials.

**Table 3:** The Nine Intraverbal Questions (three sets of three) Taught to each Participant as Listener Behavior and Assessed for Emergence as Intraverbal Behavior During Probing

	Participant 1	Participant 2	Participant 3	Participant 4	Participant 5	Participant 6
Set 1	When do you eat breakfast? When can you go sledding? When do you go to bed?	When does Santa come? When do firefighters come? When do you go to bed?	When does school end? When can you go sledding? When do you go to bed?	When does school end? When can you go sledding? When do you go to the dentist?	When do you go to the doctor? When can you go skiing? When do you go to bed?	When do the police come? When do you use crayons? When do you use toothpaste?
Set 2	When do we go to the doctor? When you use toothpaste? When you have a holiday?	When do you eat breakfast? When do you go to the beach? When do have a flag on the table?	When do you get up in the morning? When do you go to the beach? When do you have a flag on the table?	When do you call the firefighters? When do you sing the birthday song? When do you use a band aid?	When do you eat breakfast? When do you go to the dentist? When do you use a raincoat?	When do you sleep? When do you wake up? When do you wear swimwear?
Set 3	When do you wash your hands? When does school end? When do you go to the dentist?	When do you go to the store? When do you use a raincoat? When do you go to the dentist?	When do you go to the store? When do you use a raincoat? When do you go to the dentist?	When do you go to the store? When do you use a raincoat? When do you wash your hands?	When can you wear shorts? When is your birthday? When do you go to the hairdresser?	When can you wear shorts? When do you call firefighters? When do you wear a seatbelt?

### Dependent Variables and Experimental Design

During intraverbal probes, a correct response was scored if the targeted intraverbal response was emitted within 5 s of presentation of the verbal antecedent. All other responses were scored as incorrect. Responses containing the correct noun were considered correct. For example, if the participant responded "night" rather than "at night" for the question "when do you go to bed," this was scored as a correct response. During listener training, a correct response was scored if the corresponding comparison stimulus was touched within 5 s of presentation of the verbal antecedent. All other responses were scored as incorrect.

A multiple-baseline-design across behaviors (stimulus sets) was used to assess the effects of the listener training on the emergence of intraverbal behaviors. For each participant, a total of nine different unmastered when-questions were identified and assigned randomly into three sets (set 1, set 2, and set 3) with three questions in each set. Questions were chosen in collaboration with each participant’s class teacher and were included in the study because they were developmentally appropriate and consistent with the educational programs and objectives of each participant. Table 4 shows the questions used for each participant.

**Table 4:**
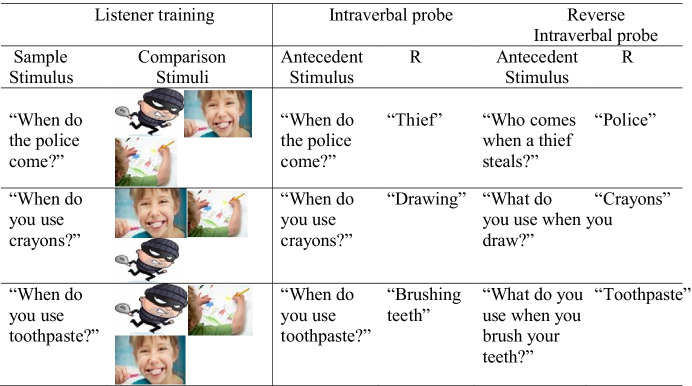
Example of Sample and Comparison Stimuli used during Listener Training and the Verbal Antecedent and Response during Intraverbal and Reverse Intraverbal Probes (Participant 6, Set 1).

### Interobserver Agreement and Treatment Integrity

Interobserver agreement (IOA) was calculated by dividing the number of agreements by the number of agreements plus disagreements and multiplying by 100. IOA data were collected by an independent observer in 38.2 % of randomly selected sessions across all participants (range, 17% to 75 %) and across all phases. Mean IOA across all phases and all participants was 100%.

Treatment integrity data were collected by an independent observer during all phases of the study and was calculated by dividing the number of correct steps by the number of correct plus incorrect steps and multiplying by 100. Data were collected on presentation of the antecedent stimuli and on the reinforcement procedure. During probes for emergent intraverbals and reverse intraverbals, correct antecedent stimulus presentation was scored if no visual stimuli occurred and if the correct question was presented. Correct reinforcement was scored if neutral verbal feedback was provided for probe trials and if praise and tokens were provided for correct responses for previously mastered intraverbal responses. During listener training, correct antecedent stimulus presentation was scored if the visual stimuli were presented correctly and if the correct question was asked. Correct reinforcement was scored if praise, tangibles, and tokens were provided for correct responses, verbal encouragement for incorrect responses, and praise for prompted responses. Treatment integrity data were collected in 28.7 % of randomly selected sessions across all participants (range, 22% to 50 %), and mean treatment integrity across all phases and all participants was 99.1 % (range, 94.4% to 100 %).

## Procedures

### Intraverbal Probes

#### Pre-Listener Training Intraverbal Probes

The participant and teacher sat opposite one another. No visual stimuli or teaching materials were present. Each participant was asked all nine questions (three questions within each set). Each question was presented once unless an incorrect response occurred. If so, the question was repeated, and scored as correct if the participant responded correctly on the second trial. If responses on both trials were incorrect, the question was scored as incorrect.

The three questions within each set were presented in a random order, and mastered intraverbals were interspersed approximately every three trials (e.g., social questions, such as, “what is your name,” “how old are you,” and “what is your mom’s name”). Correct intraverbal responding to the mastered questions resulted in praise (e.g., “Well done!”) and provision of a token. Responses to the probe questions (correct or incorrect) resulted in verbal encouragement such as “nice try” and “okay” in an attempt to minimize reactivity by avoiding differential reinforcement of correct responses and to reduce the probability of extinction, which we predicted would be more likely if no vocal consequences were given.

#### Post-Listener Training Intraverbal Probes

Mastery of listener training (see below) was immediately followed by the intraverbal probe sessions, which were identical in format to the pre-listener training intraverbal probes. An intraverbal response was considered emergent if it was absent during pre-listener training intraverbal probes and occurred correctly on post-listener training intraverbal probes.

### Reverse Intraverbal Probes

#### Pre-Listener Training Reverse Intraverbal Probes

A probe session for reverse intraverbal behavior was conducted following the first pre-listener training intraverbal probes session. The format of these probe sessions was identical to the intraverbal probes, but each question was asked in the opposite direction. For example, if, in the intraverbal probe sessions the participant answered the question, “when do you eat” with the vocal response, “when I am hungry”, a reverse intraverbal probe was, “What do you when you are hungry?” A correct response was, “I eat.”

#### Post-Listener Training Reverse Intraverbal Probes

After listener training, a probe session for reverse intraverbal behavior was conducted following mastery of one post-listener training intraverbal probe session (i.e., a minimum of eight out of nine correct responses). These probes were identical to the pre-listener training reverse intraverbal probes.

### Listener Training

Discrete trial teaching and discrimination training were used (Eikeseth et al., 2014). Picture stimuli were presented to the participants in a field of three and placed in a line across the center of the table (left, middle, right). Training sessions were conducted during each participant’s scheduled therapy sessions. Trials began with the presentation of the question, and the participant was given 5 s to respond. The order of presentation of each when question was randomized in each session, and each question was presented three times per session. The position of each picture stimulus was randomized, and each picture functioned as both an S+ and an S- in each array. Correct responses were followed immediately by putative reinforcers in the form used during each participant’s teaching sessions (e.g., praise, tokens, tangibles). Putative reinforcers for correct responses were provided for touching the correct picture, regardless of whether the participant also correctly or incorrectly tacted the picture. General praise was always delivered when other putative reinforcers were provided, but praise did not contain the label shown in the picture. For all but one participant, stimuli used as putative reinforcers were identified by interviewing the child’s primary therapist. For participant 6, putative reinforcers were identified by a multiple stimulus without replacement (MSWO) procedure (DeLeon & Iwata,^1996^).

Contingent on an incorrect response, brief verbal encouragement was provided (e.g., “good try”), and error correction was used. During error correction, the trial was repeated with a point prompt. On the subsequent trial, the same question was repeated without a prompt.

Mastery criterion during listener training was eight out of nine questions correct across two consecutive sessions. Initially, correct responses were reinforced on a fixed-ratio 1 (FR 1) schedule. After mastery, the reinforcement schedule was thinned to a variable-ratio 3 (VR 3) schedule, and following mastery on the VR 3 schedule, intraverbal probe and reverse intraverbal probe sessions were conducted.

Following listener training, if less than eight of the nine intraverbal probes were answered correctly on two consecutive probe sessions, listener training was reinstated for a second time. This was done to examine the possibility that failure to show emergent intraverbal responses was due to extinction of the listener responding. Following the second listener training, if less than eight of the nine intraverbal probes were still answered correctly on two consecutive sessions, then intraverbal training was introduced.

### Intraverbal Training

Intraverbal training was identical to the intraverbal probes except that a visual prompt was provided within 1 s of presenting the antecedent verbal stimulus. For example, if the verbal antecedent stimulus was, “when does Santa come,” a picture denoting Christmas was shown to the participant within 1 s of presenting the question. After a prompted trial, the same trial was repeated without the prompt; whenever a correct response occurred, a new question was presented. Mastery criterion was eight out of nine questions correct across two consecutive sessions. Initially, correct responses were reinforced on a FR 1 schedule. After mastery, the reinforcement schedule was thinned to a VR 3 schedule, and following mastery on the VR 3 schedule, intraverbal probe sessions were conducted.

## Results

**Fig. 1:**
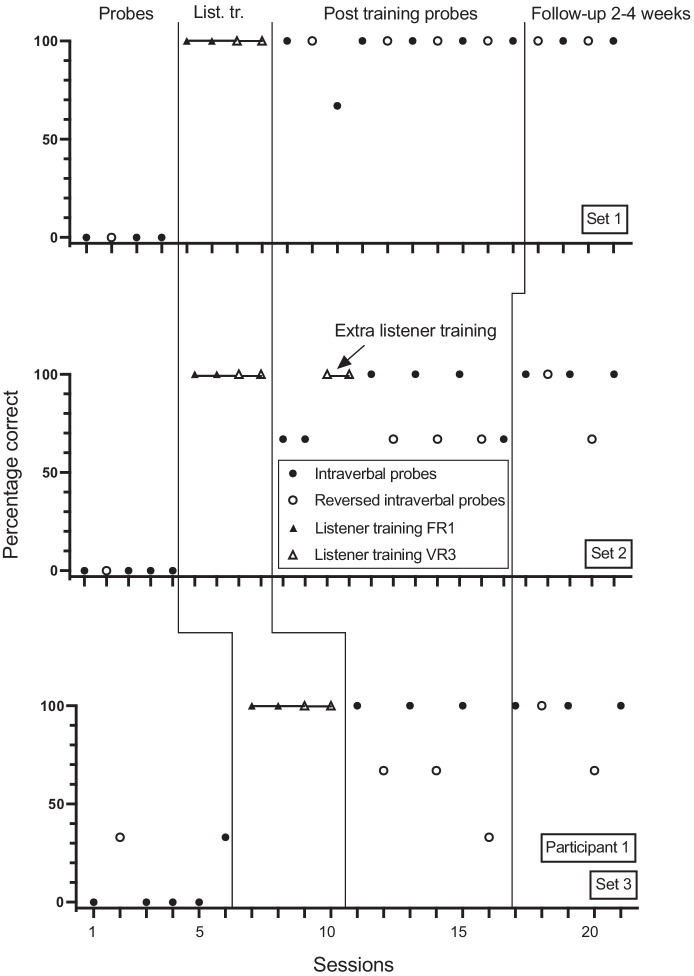
Participant 1. Percentage Correct Intraverbal Responses Pre-Training, Percentage Correct Listener Responses During Listener Training, Percentage Correct Intraverbal Responses Post Listener Training and at Follow-up, and Percentage Correct Reverse Intraverbal Responses. List. Tr. = Listener training.

**Fig. 2:**
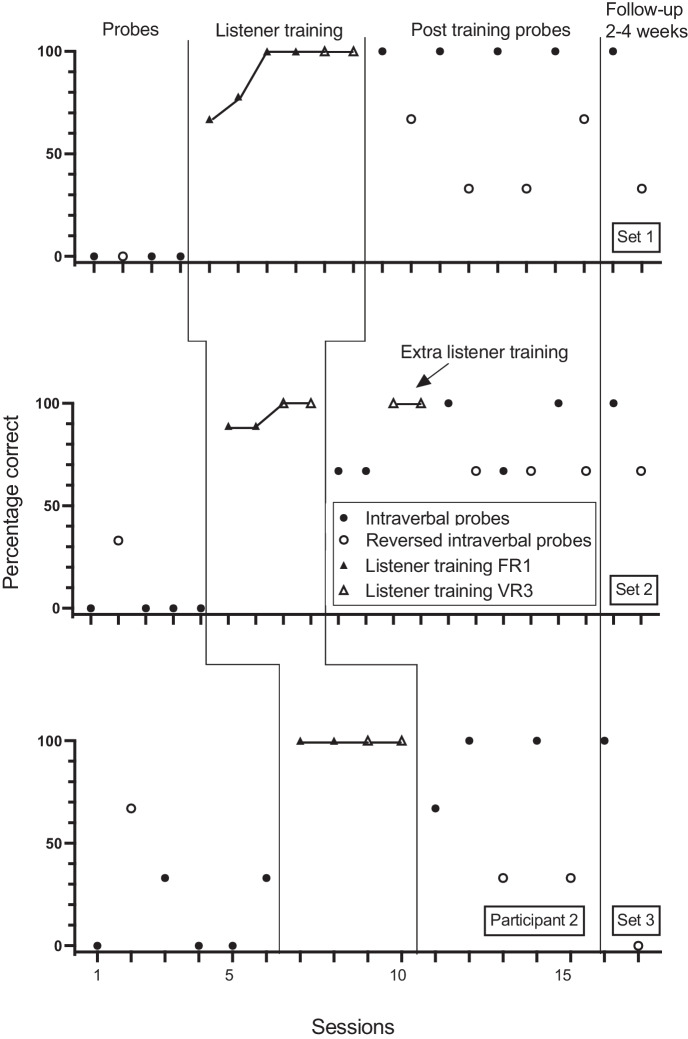
Participant 2. Percentage Correct Intraverbal Responses Pre-Training, Percentage Correct Listener Responses During Listener Training, Percentage Correct Intraverbal Responses Post Listener Training and at Follow-up, and Percentage Correct Reverse Intraverbal Responses

**Fig. 3:**
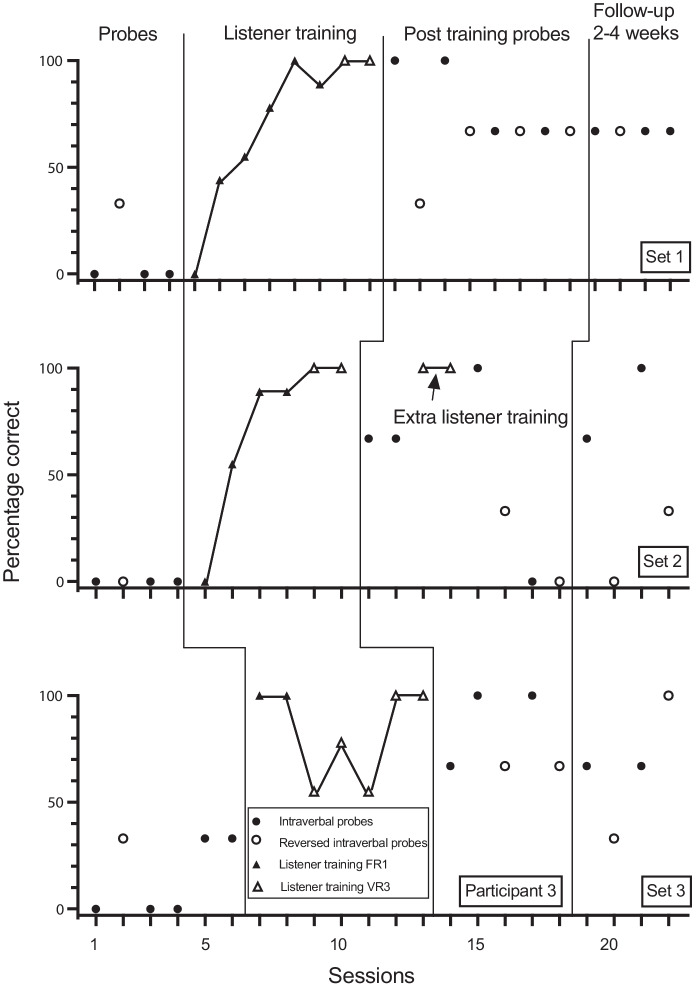
Participant 3. Percentage Correct Intraverbal Responses Pre-Training, Percentage Correct Listener Responses During Listener Training, Percentage Correct Intraverbal Responses Post Listener Training and at Follow-up, and Percentage Correct Reverse Intraverbal Responses

**Fig. 4:**
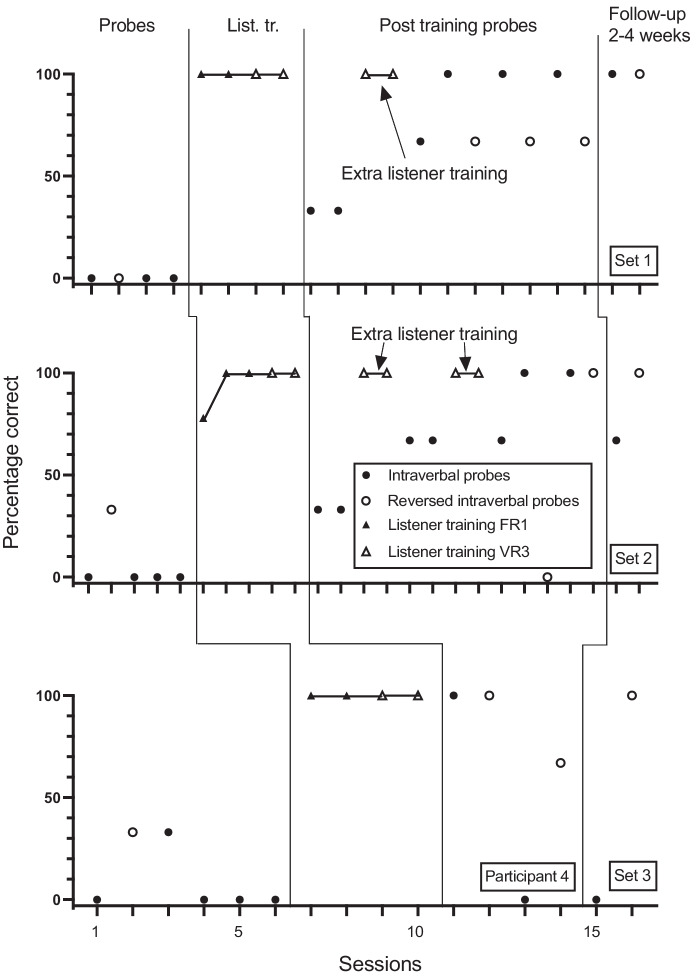
Participant 4. Percentage Correct Intraverbal Responses Pre-Training, Percentage Correct Listener Responses During Listener Training, Percentage Correct Intraverbal Responses Post Listener Training and at Follow-up, and Percentage Correct Reverse Intraverbal Responses. List. Tr. = Listener training

**Fig. 5:**
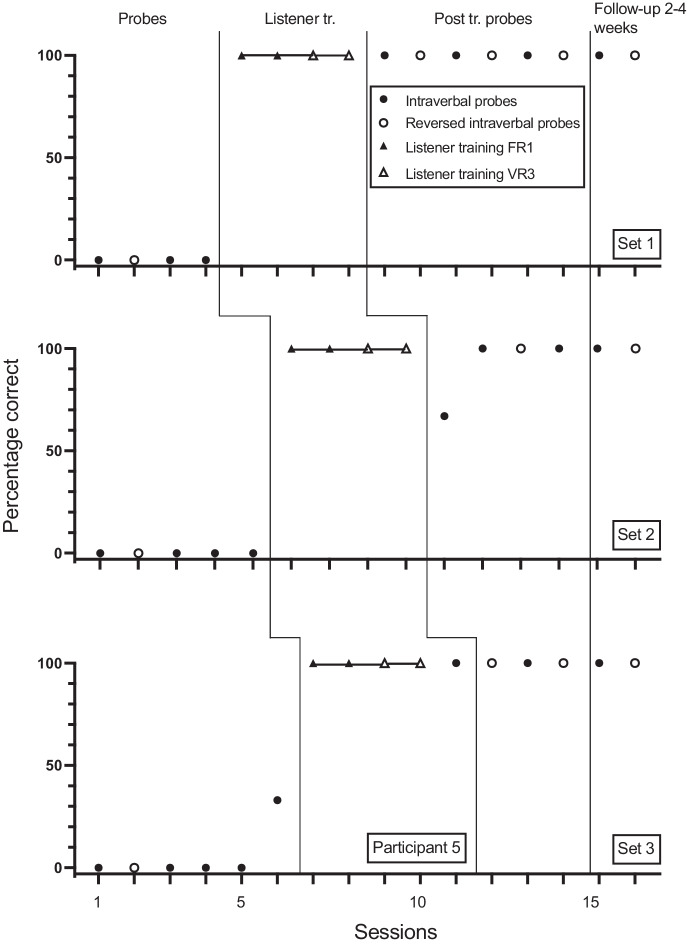
Participant 5. Percentage Correct Intraverbal Responses Pre-Training, Percentage Correct Listener Responses During Listener Training, Percentage Correct Intraverbal Responses Post Listener Training and at Follow-up, and Percentage Correct Reverse Intraverbal Responses. Listener. tr. = Listener training. Post tr. Probes = Post Training Probes

**Fig. 6:**
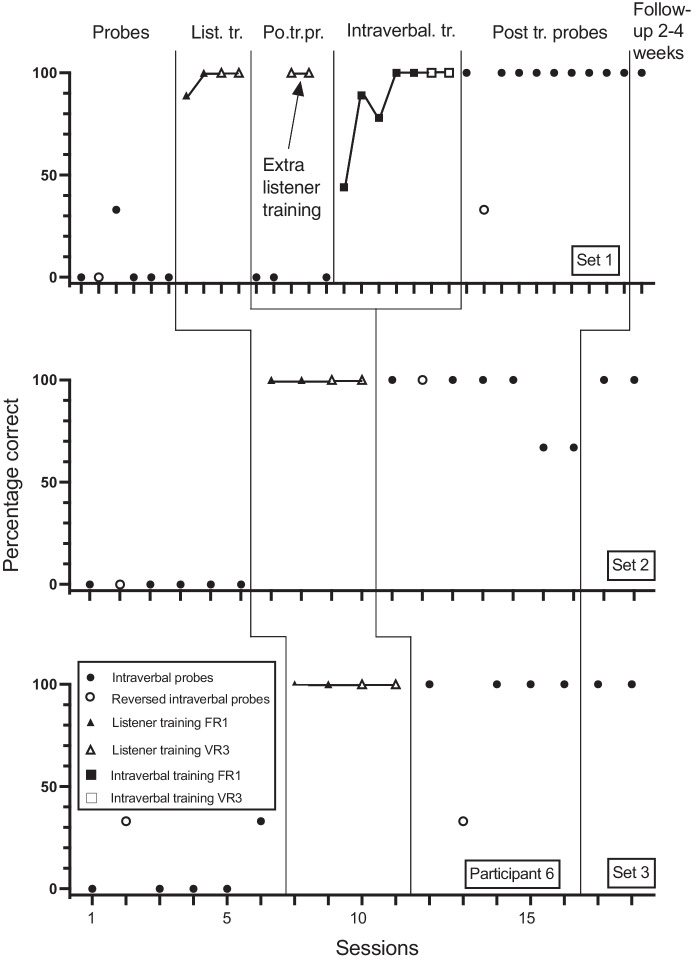
Participant 6. Percentage Correct Intraverbal Responses Pre-Training, Percentage Correct Listener Responses During Listener Training, Percentage Correct Intraverbal Responses Post Listener Training and at Follow-up, and Percentage Correct Reverse Intraverbal Responses. List. Tr. = Listener Training; Po. tr. Pr.= Post Training Probes

Figures 1, 2, 3, 4, 5 and 6 show the results of the baseline intraverbal probes (including reverse probes), listener training, post listener training intraverbal probes (including reverse probes), and follow-up intraverbal probes, for each participant. As shown in Figure 1, participant 1 answered none of the questions in the first and second sets correctly in baseline, but he answered one of the questions in the third set correctly. The participant underwent listener training in four sessions without making any errors. Following listener training, correct intraverbal responding to set 1 questions rose to 100% for 7 of 8 sessions, to 100% for 6 of 9 sessions for set 2, and to 100% for all 6 sessions for set 3. During baseline, participant 1 answered none of the reverse intraverbal probes correctly for sets 1 and 2 and one question correctly for set 3. Correct responding rose to 100% for six sessions for set 1 following listener training and intraverbal probes. For set 2, correct responding rose to 67% (2 out of 3 questions) correct for 4 of 5 sessions and 100% correct for 1 session. For set 3, correct responding increased but was variable across sessions (range, 33% to 100%).

Participant 2 answered none of the first or second sets of questions correctly in baseline but answered one question in the third set correctly (Figure 2). Listener training was conducted in six sessions for set 1 and four sessions for sets 2 and 3. Following listener training, correct intraverbal responding to set 1 questions rose to 100% for all 5 sessions, to 100% for 3 of the 6 sessions and 67% (2 out of 3 questions correct) for the remaining three sessions for set 2, and to 100% for 3 of 4 sessions for set 3. Participant 2 answered none of the reverse intraverbal questions correctly in baseline for set 1, one of the questions correctly for set 2, and two of the questions correctly for set 3. Correct responding following listener training and intraverbal probes rose to 33% to 67% correct for set 1. Correct responding for set 2 rose to 67% in four sessions. Correct responding for set 3 decreased from 67% correct in baseline to 33% correct across two sessions.

Participant 3 answered none of the offset 1 and 2 questions correctly in baseline but answered one question in set 3 correctly (Figure 3). Listener training was conducted in seven sessions for sets 1 and 3, and five sessions for set 2. Following listener training, correct responding increased with some variability. All questions in set 1 were initially answered correctly but reduced to just over 67% correct in the five subsequent sessions. For set 2, 100% correct responding occurred in two sessions, 0% correct responding occurred in one session, and 67% correct responding occurred in three sessions. For set 3, correct responding varied between 67% to 100%. Participant 3 answered one reverse intraverbal probe correctly in baseline for set 1, none correctly for set 2, and one of the questions correctly for set 3. Following listener training and intraverbal probes, correct responding occurred in 33% to 67% of trials for set 1. Correct responding for set 2 rose initially to 33% but then varied from 0% to 33% thereafter. Correct responding for set 3 varied from 33% to 100%, with 100% correct during the final sessions.

Participant 4 answered none of the set 1 and 2 questions correctly in baseline but answered one question in set 3 correctly (Figure 4). Listener training was conducted in four sessions (with no errors made) for sets 1 and 3, and in five sessions for set 2. Following listener training, correct responding increased, with some variability between sets. Correct responding for set 1 increased across six sessions (including two sets of extra listener training) and remained at 100% for the last three sessions. Correct responding for set 2 showed the same pattern, increasing to 100% over nine sessions (including four sets of extra listener training) and remained at 67% to 100% correct thereafter. For Set 3, 100% correct responding for the first session reduced to 0% for the second and third session. Participant 4 answered none of the reverse intraverbal probes correctly in baseline for set 1 and one question correctly for sets 2 and 3. Following listener training and intraverbal probes, correct responding for set 1 rose to 67% to 100%. For set 2, correct responding was 0% correct in the first session and then 100% correct thereafter. For set 3, correct responding ranged from 67% to 100%.

Participant 5 answered none of set 1 and 2 questions correctly and one of set 3 questions correctly in baseline (Figure 5). The participant underwent listener training in four sessions without making any errors. Following listener training, correct responding rose to 100% for set 1, to 100% for 4 of the 5 sessions for set 2, and to 100% for set 3. Participant 5 answered none of the reverse intraverbal probes correctly in baseline for any of the sets. Following listener training and intraverbal probes, correct responding increased to 100% for all three sets.

Participant 6 answered none of the set 1 and 2 questions correctly in baseline but answered one of the set 3 questions correctly (Figure 6). Following two repetitions of listener training for set 1, none of the intraverbal responses were correct. Following intraverbal training, correct intraverbal responding rose to 100%. For set 2, correct responding ranged from 67% to 100% without additional intraverbal training. For set 3, correct responding rose to 100%. Participant 6 answered none of the reverse intraverbal probes correctly in baseline for sets 1 and 2 and answered one of the questions correctly for set 3. Following multiple listener training sessions, intraverbal training, and intraverbal probes, correct responding increased to 33% for set 1, 100% for set 2, and 33% for set 3.

## Discussion

The present study replicated and extended Smith et al. (2016) by examining whether intraverbal responding to “when” questions emerged following listener training in children with ASD. Results showed that intraverbal responses emerged over baseline levels across all three sets of questions following listener training for five of six participants, but only one participant showed correct performances on all nine intraverbals after listener training. The remaining participants required additional listener training for one (and in one case two) stimulus sets before the intraverbals emerged at perfect (100%) levels. Participant 6 differed from the other five participants in that he required intraverbal training for set 1 before the intraverbals emerged for that set. Subsequently, emergence of intraverbal responding occurred following listener training for sets 2 and 3.

Emergent intraverbal responding can be traced directly back to the history of reinforcement in listener training (Horne & Lowe, 1996; Palmer, 2016). Results from the present study show that listener responding facilitated the emergence of intraverbal behavior but is does not show empirically how this transfer came about. Conceptually, joint control (Lowenkron, 2006) may be useful in describing how this transfer occurred. Because participants were able to tact all stimuli involved in the listener training, it is possible that the listener training evoked both a listener response and a tact. The tact behavior may have occurred covertly or overtly. For example, perhaps touching the picture denoting toothpaste evoked the tact, “Toothpaste.” If so, the touching behavior and the tact were both reinforced in the presence of the verbal antecedent stimulus (/What do you use when you brush your teeth?/). The response, “toothpaste” which began as tact may subsequently have been evoked as a self-echoic behavior. This self-echoic behavior may then have functioned as a mediating response, facilitating transfer across verbal operants (Lowenkron, 2006). The model of joint control is especially useful for understanding complex and delayed discriminations such as the one observed in the present study. This interpretation is compatible with data from participant 6, who initially required a visual prompt together with the antecedent verbal stimulus to learn the intraverbal responses. Unfortunately, data were not collected on correct or incorrect tacts that occurred during listener training, but such data would have been useful, have been reported in previous studies (e.g., Kodak & Paden, 2015), and should be considered in future studies. On the other hand, covert tacts, if they occurred, would not have been detected by such data.

We also examined whether the emergent intraverbal responses would reverse (e.g., answering the question, /When do you go to bed?/ with the vocal response, “at night” after having learned to say, “go to bed” when hearing /What do you do at night?/). Results showed that reverse intraverbal responses emerged across all three stimulus sets for three of the participants. For the remaining three participants, responding was more variable, but reverse intraverbals occurred above baseline levels for most stimulus sets. A variable that was constant across listener training and intraverbal probes was the verbal antecedents, which was identical across both conditions and included responding to when questions. This may have facilitated the transfer from listener behavior to intraverbal behavior. The antecedent for the reverse intraverbals, however, included responding to either who or what questions. Hence, the antecedent for the emergent intraverbal responses differed from the antecedent for the reverse intraverbals, and this could explain, in part, why there was more emergence of intraverbals as compared to reverse intraverbals.

To our knowledge, this is the first demonstration of reverse intraverbal responding emerging from listener training in individuals with ASD. However, the purpose of the study was not to show or to propose that listener training is the most effective way of establishing emergent intraverbal responding. Indeed, studies have shown that tact training typically is more effective in establishing intraverbal responding than listener training both in typically developing individuals as well as in individuals with ASD (e.g., Allan et al., 2015; Cortez et al., 2020; Petursdottir et al., 2008a, b; Thakore & Petursdottir, 2021). Rather, we sought to examine whether intraverbal and reverse intraverbal relations could emerge after listener training to demonstrate how behavior analysts may account for verbal behavior emerging without a direct reinforcement history.

The results of this study showed emergence of intraverbal responses as well as many instances of reverse intraverbal responding, suggesting that the intraverbal responding was not rote learning, but based on the reinforcement history in listener training. In rote learning, the intraverbal responses can be said to be under configural stimulus control (Devine et al., 2016; Pearce, 1987, 2002), in which the intraverbal response is controlled by the whole of the verbal antecedent stimulus. Under configural stimulus control, there is no independent control by the individual elements of the verbal antecedent. In the present study, because reverse intraverbals emerged, these relations could not have been established under configural stimulus control, suggesting that listener training may perhaps prevent establishing intraverbal responses under configural stimulus control. This possibility merits further research.

Also related to stimulus control, 100% correct responding occurred from the onset of listener training for participants 1 and 5. Because no errors were made and no prompts were given during listener training for these two participants, they had the listener behavior in their repertoire before entering the study. Yet, they did not respond correctly to the intraverbal probes until they were required to respond to the antecedents for the intraverbal response as a listener.

The results for participant 6 showed that listener training was not sufficient to establish intraverbal responding for set 1. Training the intraverbals using a visual stimulus as a prompt was required before the intraverbal responses occurred. The intraverbal training was identical to the intraverbal probes except that a visual prompt was presented together with the antecedent verbal stimulus. For example, if the verbal antecedent stimulus was, /When does Santa come? /, a picture denoting Christmas was shown to the participant. Hence, no echoic prompts were provided at any time during any phase of the experiment. After learning the intraverbals in set 1, intraverbal responding emerged following listener training for the two subsequent stimulus sets. This finding may suggest that intraverbal training using visual prompts may be an effective way to establish intraverbal responding when listener training alone is not sufficient. This could be explored in future studies.

The reasons why listener training was not sufficient to establish intraverbal responding for participants 6 is not clear. It is possible that the participant lacked certain key prerequisite skills, although the absence of specific prerequisites was not detected from data in the present study. Future research could examine more closely whether it is possible to identify prerequisite verbal behavior using assessments such as the Verbal Behavior Milestones Assessment and Placement Program (VB-MAPP, Sundberg, 2008) in addition to the ABLLS-R (Partington, 2010). Also, an analysis of the extent to which the participants show other potential prerequisites such being able to acquire verbal behavior through naming and joint control could be explored.

Questions also remain about sources of stimulus control established during listener training and intraverbal probes. One limitation of the current study is that we did not conduct an analysis of the stimulus control exerted by individual elements of the antecedent verbal stimulus. Future research could examine the effects of variations of the same verbal antecedent on emergence (e.g., /when do you go to bed?/ vs. /when do you go to sleep/” on the intraverbal response, “at night”).

Further, an analysis of the effects of the visual stimulus used during listener training on intraverbal emergence was not conducted as only one picture for each question was used. It may be the case that only one part of each picture (e.g., the moon included in the picture for the response, “at night”) exerted stimulus control over the response and future research could examine the effects of using a larger array of pictures during listener training to see if this facilitates emergence of the intraverbal response.

For participants 1 and 5, variation in the criterion to conduct additional probes or stop conducting these occurred. For these two participants, extra post-listener training probes and reverse intraverbal probes were conducted. Variations in decisions to continue versus stop probes is a limitation which should be corrected in future studies.

Another limitation pertains to experimental control. Performance on the reverse intraverbals trials were assessed only once during baseline. Additional baseline probes for reverse intraverbals would have provided a more convincing demonstration of experimental control, and hence, a clearer demonstration of the emergence of revers intraverbals.
